# Perceptions of People From South Asian Backgrounds With Chronic Kidney Disease on Digital Health Interventions: Patient and Public Involvement and Engagement Consultation Focus Group

**DOI:** 10.1111/hex.70745

**Published:** 2026-06-28

**Authors:** Courtney J. Lightfoot, Roseanne E. Billany, Heather Hill, Matthew P. M. Graham‐Brown

**Affiliations:** ^1^ Division of Cardiovascular Sciences, School of Medical Sciences University of Leicester Leicester UK; ^2^ Leicester Partnership for Kidney Health Research, School of Medical Sciences, University of Leicester and NIHR Leicester Biomedical Research Centre Glenfield Hospital Leicester UK; ^3^ NIHR Leicester Biomedical Research Centre Leicester UK; ^4^ Department of Renal Medicine University Hospitals of Leicester NHS Trust Leicester UK

**Keywords:** chronic kidney disease, digital health, inequalities, minority ethnic, underserved groups

## Abstract

**Introduction:**

Digital health interventions (DHIs) are increasingly popular as a potential method to address educational and support needs of individuals with long‐term conditions, as they are largely accessible to most people, can be highly effective, and delivered at low cost. We have developed a DHI to better support chronic kidney disease (CKD) self‐management and demonstrated its efficacy in a UK‐based clinical trial. The trial population, however, was fairly homogeneous, and those from underserved populations (including ethnic minority groups), who would have likely benefited from the DHI, were underrepresented. Understanding how ethnic minority communities perceive DHIs is essential for identifying their needs, priorities and challenges, and for ensuring that future DHIs are relevant, acceptable and equitable. Given the potential impact of DHIs, patients should be actively involved in their design and implementation. To support this, patient and public involvement and engagement (PPIE) consultations were conducted to: 1) understand people with CKD from South Asian (SA) backgrounds’ perspectives of DHIs; 2) identify factors that require consideration when adapting DHIs to address health inequities; and 3) clarify how best to conduct PPIE projects in adapting DHIs for traditionally underserved groups, particularly ethnic minority groups.

**Methods:**

PPIE consultation focus groups were conducted in‐person with ten individuals (60% male, age: 69 ± 7) from SA backgrounds thrice, audio‐recorded and transcribed verbatim. Data were analysed using thematic analysis.

**Findings:**

Participants identified several barriers and facilitators to accessing and using DHIs. Language was reported to be the biggest barrier to accessing and using online health information. Having culturally appropriate information available in their own language would increase the acceptability and uptake of the DHI among SAs. Not making assumptions and being respectful about individual choices, cultural or religious beliefs, was considered key to engaging people. Promotion by a trusted person (doctors, peers, community leaders) was perceived to help alleviate the fear of DHIs and improve uptake and usage.

**Conclusion:**

People from SA backgrounds are open to using DHIs, but they need to be available in their own language and be culturally appropriate. The findings will be used to adapt our CKD self‐management DHI to better address the needs of those from SA backgrounds.

**Patient or Public Contribution:**

Patients and members of the public were directly involved in the participation of the patient and public involvement and engagement (PPIE) consultations. Community engagement officers from the Centre of Ethnic Health, who were from South Asian backgrounds, were involved in the PPIE consultation, recruitment, development of a semi‐structured focus group guide, and facilitation of consultation focus groups.

## Summary

Digital health tools are becoming an important way to help people manage long‐term health conditions because they are easy to access, cost‐effective, and can provide useful support. We developed a digital health intervention (DHI) to help people with chronic kidney disease (CKD) manage their condition and showed that it works well in a clinical trial. However, the trial included mostly similar participants, and people from underserved communities, who might benefit the most, were not well represented. This study looked at what makes it easier or harder for people from South Asian backgrounds to use DHIs. We held patient and public involvement and engagement (PPIE) consultation focus groups with 10 participants and analysed their views. The biggest barrier was language, and participants said they would be more likely to use DHIs if information was available in their own language and reflected their cultural needs. They also felt that recommendations from trusted people, such as doctors or community leaders, would help build confidence. These findings will guide changes to make our CKD DHI more inclusive.

## 1 Background

Digital health interventions (DHIs) have gained increasing recognition as a means to increasing access to health information and services, improving cost‐effectiveness and empowering people to take control of their health [1]. Whilst DHIs are often appealing due to their potential to deliver low cost at scale, the implementation attempts of such interventions can be challenging [2]. Consequently, there are a limited number of successful interventions beyond the pilot or feasibility stage [2]. We have developed a theory‐ and evidence‐based DHI to support people with chronic kidney disease (CKD) to better self‐manage their condition [3], called My Kidneys & Me (MK&M), which has demonstrated positive effects on patient activation (knowledge, skills, and confidence in managing their own health [4]), self‐management knowledge and behaviours [5]. The findings from the SMILE‐K trial, which evaluated MK&M, have some generalisable limitations due to the high proportion of White participants and may not be generalisable to those from minority ethnic groups [5]. Due to the nature of the trial and DHI, those with limited digital, health, and English literacy were likely unable to participate.

People from disadvantaged and underserved groups (defined as populations with limited access to healthcare who face substantial global health disparities arising from multiple intersecting factors [6]), including those from lower socio‐economic groups and from ethnic minority groups, are more likely to develop CKD, progress faster towards kidney failure, and die earlier with CKD [7]. Consequently, these individuals have poorer CKD‐related health outcomes and are less likely to engage with healthcare teams or self‐management strategies. This may, in part, be due to low health literacy (a social determinant of health associated with health‐related disparities [8, 9]), which can negatively impact disease self‐management and health behaviours [10]. These patients, therefore, potentially have the most to gain from a DHI like MK&M. Given that strategies for the self‐management of a long‐term condition (LTC), like CKD, might vary based on socio‐cultural and economic status [2], it is clear that revisions are required to the MK&M DHI to better support people from ethnic minority and lower socio‐economic groups to self‐manage.

People from South Asian (SA) backgrounds are at increased risk of chronic kidney disease (CKD), with a higher incidence compared with other ethnic groups [11]; they are more likely to develop CKD at a younger age and experience more rapid disease progression, leading to end‑stage kidney disease (ESKD) [7, 11]. South Asian individuals with CKD are more likely to be younger, male, socioeconomically deprived and to live in an urban area, and to have more advanced CKD [11]. In the UK, people from Asian backgrounds make up the second‐largest population (approximately 9%), with most individuals of South Asian origin (approximately 7.5% of the UK population) [12]. Notably, Leicester is one of the most ethnically diverse cities, with a majority‐minority population, with almost half (43.5%) of its population identifying as Asian and 40% reporting South Asian heritage [13, 14, 15]. Leicester is also ranked the 12th most deprived area in the UK [16].

Before adapting MK&M, we conducted a series of patient and public involvement and engagement (PPIE) consultations to try to understand key informants’ perspectives and experiences of DHIs. Understanding how people from ethnic minority and lower socio‐economic groups perceive, access and use DHIs is important to identify barriers and enablers to engaging with DHIs.

## 2 PPIE Consultations

Digital health technologies have the potential to support better health; however, they risk exacerbating inequalities by disproportionately benefiting a subset of the population [17]. Those who stand to benefit most from the digital health transformation may be excluded or disadvantaged [18], as a result of widespread implementation, which could introduce additional social and equity implications [19, 20]. Research has shown that people's individual characteristics can lead to differences in access and use [17]. We are interested in how people from ethnic minority communities view DHIs to identify needs, priorities, and challenges of these individuals to ensure that our research and its design are relevant. Thus, we conducted a series of PPIE workshops or consultations with members of the public to discuss and provide feedback on research. The findings from this work will offer valuable insight into how people from underserved groups perceive digital health interventions (DHIs). This will help identify key considerations for developing and adapting DHIs that better meet their needs. Insights from this PPIE consultation will inform future research to support and sustain digital inclusion, an essential step toward achieving digital equity.

## 3 Aims

We conducted this consultation to begin to understand the factors to consider when adapting a DHI for a South Asian population, as well as the methods for achieving these adaptations. This work was exploratory and sought to enable meaningful engagement with patient and public contributors to identify priorities, concerns, and experiential knowledge that may not be captured through traditional research designs. These consultations aimed to: 1) understand people with CKD from South Asian (SA) backgrounds’ perspectives of DHIs; 2) identify factors that require consideration when adapting DHIs to address health inequities; and 3) clarify how best to conduct PPIE projects in adapting DHIs for traditionally underserved groups, particularly ethnic minority groups.

## 4 Methods

### 4.1 The Consolidated Criteria for Reporting Qualitative Research (COREQ) [27] to Elaborate on Our Methodology

Setting

This PPIE consultation was conducted in Leicester, UK, one of the most diverse cities in England, where there is a high population of individuals from South Asian backgrounds, with 43% of individuals identifying their ethnic group as Asian [15]. Leicester is also home to the Centre of Ethnic Health Research (CEHR), which provides support to understand the needs of ethnic minority and underserved communities when planning and undertaking research and healthcare delivery [21]. All sessions were held in person at a local community centre that was central and convenient for participants; the venue was chosen by the participants. The dates and times of the sessions were mutually agreed upon based on project timelines and participants’ availability.

### 4.2 Public Collaborators and Recruitment

A community engagement officer from the CEHR met with the lead researcher (CJL) to discuss the project in detail and expectations. The CEHR community engagement officer already had well‐established links and relationships with leaders of South Asian community groups within and around the city of Leicester. The lead researcher attended a number of community events with the community engagement officer to provide some information about the project, including a summary of the work, participant requirements and expectations. In addition, the researcher provided some information about general kidney health and lifestyle in the form of the “Your Kidneys and Your Health” information booklet [3].

Individuals from the South Asian community groups who reported living with chronic kidney disease (Stages 3‐4 (eGFR: 15‐60 ml/min/1.73 m^2^) who expressed an interest were invited to participate. Participants were selected based on their heritage, including their ethnicity, religion, and cultural background, to ensure a variety of perspectives and experiences. Per NIHR guidelines, participants were remunerated at £25/hour, received refreshments and had their expenses reimbursed.

### 4.3 PPIE Consultation Structure

A combination of PPI and focus group methodologies were used to explore individuals’ perceptions, experiences, and understandings of DHIs [22]. A series of semi‐structured focus group topic guides, with open‐ended deductive and inductive questions, were developed to allow exploration of any issues or subjects of interest from the discussion, whilst allowing flexibility to probe topics and clarify understanding. Topic guides were reviewed and revised between sessions. The sessions were supplemented with a PowerPoint presentation, highlighting key questions and prompts, to facilitate discussion and ensure progression. A total of ten individuals from SA backgrounds, with (multiple) LTCs, were invited to take part in three sessions, across a 3‐month period.

The groups were led by CJL and facilitated by the CEHR community engagement officer. A note‐taking facilitator was also present. Sessions were held predominantly in English (requiring individuals to have a level of English proficiency to participate), but the community engagement officer translated information into participants’ own languages where necessary to improve understanding, support the articulation of participants’ responses, and minimise any potential language barriers. Prior to the initial session, participants provided written informed consent to contribute to the sessions’ recordings. All participants were encouraged to share their thoughts and feelings; the lead researcher (an experienced qualitative researcher) and the community engagement office (who had established relationships with the participants) created an inclusive environment, ensuring that each participant had the opportunity to contribute by actively encouraging quieter members to participate in the discussion.

Consultations were structured as follows:

1. general introduction and explanation of ground rules;
2. semi‐structured group discussions, sharing of initial thoughts and ideas of DHIs
3. presentation by CJL defining and explaining DHIs and their use in healthcare
4. semi‐structured collective group discussion building on CJL's presentation and initial thoughts and ideas.

We treated group discussions as focus groups to take advantage of the social context of discussing ideas in a group format. This allows individuals to “reflect and refine” their insights into perspectives [23]. Initial discussions were held before the presentation to capture unrefined thoughts and instinctive reactions of DHIs, unaffected by formal explanatory input, and were later expanded through group discussion after the presentation.

### 4.4 Analysis

Consultations were audio‐recorded and transcribed verbatim using professional transcription services. Data were analysed using thematic analysis [24], which was led by one researcher (CJL). Information power was considered to be of greater importance than data saturation, thus an information‑rich sample was prioritised over a large one [25].

The first stage of analysis involved developing a coding manual and organising segments of similar or related text to assist with interpretation. The next stage involved summarising the data, outlining key points, and identifying potential themes. Following this, the codes were applied deductively (derived from the study aims and relevant literature/theory) and inductively (developed through close engagement with the dataset), and themes were revisited, revised, and reorganised into more appropriate groups. Finally, the coded themes identified were corroborated by the other researchers. An abductive approach was used to combine codes to create clusters of ideas or similar overarching themes. The themes identified summarised key discussions across all the focus groups. Quotes were extracted from the transcripts and linked to appropriate codes and themes. Quotations and descriptive excerpts that best illustrated these themes were selected from individual transcripts and consolidated to form cohesive narratives that authentically reflected the discussions across the focus groups.

### 4.5 Ethical Considerations

This work involved PPIE consultations; therefore, ethical approval was not necessary, as it is not considered mandatory for PPI in the UK per NIHR guidance [26]. However, as stated above, individuals provided written informed consent to participate in the focus group to reflect ethical practices [26].

## 5 Results

### 5.1 PPIE Participants

This group of individuals participating in the consultation sessions is not intended to be representative of the Leicester South Asian community in general but rather represents a subset interested in collaborating on research. They contributed multiple layers of perspective informed by their varied experiences. We collected basic demographic data to provide context for understanding participants’ identities and backgrounds. Participants (60% male, mean age: 69 ± 7) were from a range of education levels (completed primary school, n = 1; high school, n = 5, college, n = 1; university, n = 3), ethnicities (70% Indian, 30% Pakistani) and religions (Islam, Hinduism, Sikhism).

Participants varied in their knowledge and experience of searching for health information online, digital health systems, technologies and DHIs. These include 1) most with experience of using online systems for booking appointments and ordering prescriptions; 2) some with pre‐existing knowledge and/or experience of searching for health information online; 3) some with pre‐existing knowledge and/or experience of DHIs available.

### 5.2 Themes

Through iterative analysis, four primary themes were identified, with corresponding subthemes.

### 5.3 Primary Theme 1: Challenges of DHIs

The first theme related to PPIE members’ perceptions and experiences of accessing and using DHIs in health care. It focused on three subthemes: 1) accessibility and usability; 2) understanding the information provided; and 3) trust in healthcare providers and information.

### 5.4 Accessibility and Usability

PPIE participants described accessing health information online as increasingly convenient, noting that the digital environment has made resources more accessible, influential, and cost‑effective. They valued the portability and ease of digital materials across settings, viewing them as more practical than physical booklets, which are easily misplaced or overlooked.

I think now we are living in the digital world now, and moving forward in the digital world, and digital is the best media, it's more accessible and more money‐saving as well. Plus, I mean how many booklets you could carry or read? On the digital, I mean where I am, I'm in the bus, aeroplane where I am, I can access the information whenever I want it, otherwise I might get a book from you but I could read it one time, put it away and forget it.

Digital formats were also described as easier to read, understand, and share, but acknowledged that not everyone feels confident using them. They stressed the need for support for those facing digital barriers and emphasised that tools must be user‑friendly, with simple, streamlined navigation to encourage engagement and progression through a programme.

Digital it's more accessible and more easy to read and understand. I think digital is the most effective. And reach more and more people. A lot of people, they can't use digital media but we should try to help them in whatever way we can. It should be user‐friendly for them. I think if we want to do a programme, I think it has to respond to need. To be very free of problems and a simplified way of going from one step to another so that nobody gets stuck and they feel encouraged to go further and achieve their goal.

### 5.5 Understanding the Information Provided

PPIE participants stressed the need for people to genuinely understand the health information they receive to make informed decisions. They noted wide variation in literacy, language ability, and digital skills; while some individuals feel confident finding and evaluating online information, others, particularly those facing language barriers, limited digital skills, or unreliable access, struggle to locate, comprehend, or use the information they need.

Linguistically it's making sure they understand what's happening in their body and understand the treatment they're going to receive, the side effects, things like that so it's very important that you get – sometimes interpreters may be – you can use family but it's not always appropriate. Some families sometimes select what they can and cannot tell their loved one. The person then hasn't got a choice, the person who's the important person, the patient, then they lose that choice don't they or they haven't got the information to make an informed choice. If that person can communicate all that information that's on that programme in a correct and accurate way in either the language that they speak.

Linguistic accessibility was stressed as essential for understanding one's condition, treatment options, and potential side effects, noting that relying on interpreters or family members can be unreliable or inappropriate. To support informed decision‑making, they advocated for accurate information in a person's preferred language and for multimodal formats, such as audio paired with subtitles and clear, meaningful images, to aid those with low literacy or limited language proficiency.

Audio is the best option ‐ you're listening, you know what they're saying – and have the subtitles on ‐ that's the easiest, so you know what is saying, what is going on. Sometimes people use subtitles but then again if you can't read the language then that's not going to be – that's going to be another path that's really not going to be effective really, yeah I possibly say it can have or some of them can be better pictures than that, because not everyone understands. Pictures need to make it more clear.

### 5.6 Trust in Healthcare Providers and Information

PPIE participants described complex, often historically rooted issues of trust in healthcare within some South Asian communities. Concerns about confidentiality in close‐knit networks may make some individuals less comfortable sharing sensitive information with professionals from their own ethnic background, fearing it could be shared within the community. Some also perceived White doctors as more educated, authoritative, or impartial, views acknowledged as generational and tied to past social hierarchies rather than actual differences in competence, and not reflective of younger generations. These discussions highlight the need for culturally sensitive practice and for building trust through clear communication, reassurance about confidentiality, and inclusive professional representation.

They will trust any professional, but they will all trust a white professional more – I don't know why. It's strange isn't it, but it's true, a lot of people do – lots of issues why South Asians would trust a white person as professional and they would appreciate their education. White people have more knowledge, and more experience than us. I just think it's something to do with – I think it's generational. It's to do with trust, people don't trust. So say if you have an Asian family and they've got an Asian social worker, they won't trust that social worker because they're afraid of confidentiality – they think that whatever they're going to say is going to get into the community. So, preferably a white doctor, not a non‐White person. Because they will understand that this person has the power, he knows that he has the knowledge to do the right job. Nurses, etc., could be any colour or creed. It's just something that we have brought from our olden days.

### 5.7 Primary Theme 2: Considerations for Developing or Adapting DHIs for Different Ethnic Minority Groups

The main subthemes that emerged regarding factors that need to be considered when developing or adapting DHIs for underserved groups, notably those from ethnic minority groups, included: 1) multilingual approach; 2) culture and religion considerations; and 3) diversity and meaningful representation.

### 5.8 Multilingual Approach

PPIE participants discussed the significant language barriers created when digital health information is available mainly in English, noting that the lack of multilingual resources can exclude those who do not speak or read English. They emphasised the need for a multilingual approach to ensure such tools are accessible and do not discourage use.

Going on digital means that you are being forced to learn English and the people who don't know English, they will never use it, it's as simple as that. So it is a big barrier. I haven't seen it [the NHS website] in any other language. So people who don't know English aren't going to use it.

The importance of providing information in people's preferred languages was emphasised, but the practical challenge of the many languages and dialects spoken across South Asian communities was acknowledged. They stressed that translation efforts should be guided by the needs of local populations rather than attempting to cover every possible language.

I would want it in my own language. And similar people who've said the same about their languages, I would think, different languages. So at least they can read their language. I think you have to be careful because I don't know how many languages we've got, we've got over 100 languages, spoken languages and different dialects as well – so you've really got to be careful which languages you're going to pick. You don't want to do 100 different languages and then there might only be one person who might use a certain language.

It was noted that written translations alone may not be sufficient, as some individuals cannot read even their first language; therefore, using audio formats in carefully chosen languages and clear visual content was recommended. The need for targeted, context‑sensitive linguistic support to ensure equitable access to digital health information was highlighted.

You've got to respond to need but also I think if it's done verbally, because like you said and I agree, some people can't read their first language anyway, let alone speak it. And there are some languages which have a dialect, so I think you have to respond to the need of that particular language.

### 5.9 Culture and Religion Considerations

PPIE participants emphasised the need to consider religious practices, cultural norms, and dietary traditions when providing health information. They noted that people may question whether treatments are permissible during periods of religious observance, though many faith traditions allow essential medical care and permit adaptations or postponement when necessary.

It's to do with if they're really sort of maybe religious or if there's a particular culture that they follow, whether it's appropriate for them to receive that treatment. It could be, like if it's Ramadan and if it's fasting, is it all right to have that treatment, is it safe to have it while they're fasting? I think to my mind religion doesn't stop you to have your treatment. I mean in our religion you are allowed it, even in Ramadan. If you genuinely need it, you can delay fasting afterwards.

They highlighted the need to ask respectfully about religious or personal dietary needs, given the wide variation in dietary practices within and between cultural groups. They stressed that open, non‑assumptive conversations are essential, since standard dietary advice may not suit everyone. Familiarity with a person's cultural context, combined with personalised and sensitive communication, was considered key to ensuring that health information is appropriate and acceptable.

The thing with the kidneys, I mean, because it says a special diet, you'd have to work around what that person's dietary needs were. A white person's diet to you would be completely different. It's being respectful about asking them is there anything they don't eat, maybe it's just individual choice, it could be because of religious beliefs. Just make sure you ask them what their dietary needs are. You have to think of different scenarios, then what people you'd use and the dietaries and things like that. I think like for like – familiarity is more helpful for that person.

### 5.10 Diversity and Meaningful Representation

PPIE participants outlined the importance of representation and diversity in digital and video‑based health information. They noted that using only White individuals fails to reflect the wider population and may reduce engagement among minority ethnic groups. People are more likely to trust and connect with information presented by individuals who share similar backgrounds or experiences, making diverse representation, across patients, carers, and community figures, crucial for improving relatability and credibility, with real patients seen as especially influential.

If you had a patient who was white, the professional was white, the ambassador or the whoever, champion, they were white, then that doesn't really reflect the society we live in, or the kidney society, or any other illness. You got people from all those communities. I think, just to focus on diversity as well involving people from different, you know, you could have health professionals who's white, a faith leader who's black, or you could have a patient who's black. I think in the past when people have used just, white people, people here don't feel like they can relate to anything.

Participants explained that people are more likely to connect with someone whose background, dietary practices, or lived experiences resemble their own. While they felt that healthcare professionals could be of any ethnicity without undermining trust, they stressed that patient‐facing materials should include diverse voices and imagery to ensure inclusivity and foster meaningful engagement among all communities.

They would probably prefer Asian person than listen to a white one. If there was similar person or different groups of people coming down handing the information, that would help a lot. Different backgrounds. Real people. I think that's more powerful. Because they can relate themselves to the person talking. I think healthcare professionals, I think it's different to having patients and carers who are from black and other diverse communities, but healthcare professionals, I think they can be White, I don't think people really look at it.

### 5.11 Primary Theme 3: Recommendations to Improve Uptake of DHIs in Underserved Communities

#### 5.11.1 Community Engagement and Promotion

PPIE participants emphasised the strong influence that community and faith leaders have on shaping health beliefs and behaviours, noting that many people trust these figures more than health professionals due to their cultural authority and central role in community life. When well‑informed leaders share health messages, the information is perceived to carry greater credibility, cultural relevance, and acceptance.

A lot of people, they have a lot of faith in the faith leader or your community leader and if he is properly educated about this problem then he can explain that it has more influence with the people, instead of me saying to somebody probably it doesn't work, but if you said faith leader, any person or community leader, if he say something so I then expect more effect on it. Because it's in our minds, that's how we're brought up, that's how the community is.

By introducing or endorsing health materials in ways that feel relevant to their communities, community and faith leaders could act as influential ambassadors for health interventions. Involving these leaders in familiar spaces, such as places of worship or community centres, was seen as an effective way to build awareness, trust, and confidence. Meaningful engagement with trusted community figures was considered vital to increasing the reach and impact of health interventions.

All the communities have a leader, it depends, religious leader or community leader or area leader, or whatever, so he has more influence on your mind and it depends how he explains to you. If there is a lot of influence. He or she could be the ambassador then, before the actual videos introducing, and who's saying it and what he is saying it, that's the main thing, how we say it and who it says. It's trust isn't it, they would trust somebody like that. It would raise a lot of awareness in our community. I think it's just people are just scared sometimes.

#### 5.11.2 Appropriate Digital Support

PPIE participants noted the growing reliance on digital methods for delivering health information, recognising them as efficient, cost‑effective, and less resource‑intensive than printed materials. However, they emphasised that digital delivery cannot be a one‑size‑fits‑all solution.

Digital is the only solution at the moment because you save a lot of money. When you're printing out the booklets with all the information, how much is it going to cost you? You can put it on digital and you try to approach the people who can't read it. There are some people who want to see piece of paper, to do it face‐to‐face as well. [It] would be very difficult with all the different barriers we've talked about. And very costly as well, so I think that's why people are looking to go digitally. This is a good way of doing it if you can do it.

It was acknowledged that some individuals, particularly older adults or those with limited digital skills, may struggle with digital resources and prefer face‑to‑face or printed information. The need for personalised support, such as guidance through digital platforms, to build confidence and reduce anxiety about technology was highlighted. While digital approaches were viewed as efficient, PPIE participants stressed the importance of accessible alternatives and tailored assistance to ensure no one is excluded from essential health information.

If I think of people who are older than me, if I think of my parents, they must find it really difficult. They need to see a person face‐to‐face and show them properly how to do it. I think if they have support accessing it, I don't know if ‘mentors’ is the right word but they could go and show how to do it or something to start them off. That would reassure the other person who might have a fear of technology and everything if somebody sits with them and does it with them, they can trust that person.

#### 5.11.3 Primary Theme 4: PPIE Recommendations for Developing and/or Adapting DHIs

PPIE participants provided recommendations on developing or adapting DHIs for different populations, particularly ethnic minority groups, including having community collaboration and inclusive representation in DHI development, and community‑rooted co‑design to enhance engagement with DHIs.

### 5.12 Community Collaboration and Inclusive Representation in DHI Development

PPIE participants emphasised the need for sustained, community‑centred engagement when developing or adapting DHIs, stressing early and ongoing involvement. They highlighted that trust and effective communication are built through collaboration across diverse communities, with people sharing experiences and learning from one another. Prioritising diversity in recruitment and representation was seen as essential to ensuring digital resources resonate with the populations they aim to serve. PPIE participants advocated co‑designing DHIs with community members who bring relevant lived experience, further strengthening cultural relevance and trust.

Everything works together; like they have to work together to send the message or the problem, they have to work together, combined, whatever it is. Community communication is one thing ‐ that we talked to each other and they tell you about their experiences. Focus on diversity as well, involving people from different communities, patients and carers who are from black and other diverse communities. I think it's very important to get the right mix, it's hard, but I think it's important.

### 5.13 Community‑Rooted Co‑Design to Enhance Engagement with DHI

PPIE participants explained that people are more likely to engage with DHIs when they are introduced or supported by familiar, relatable figures within their own community networks. Reaching people in familiar spaces, such as community centres and places of worship, and investing time in relationship‑building were seen as key to reducing fear and increasing awareness. They emphasised that co‑design should occur within the community, with trusted members involved throughout, to ensure DHIs reflect lived experience, align culturally, and are delivered through channels communities recognise and trust.

We need somebody like you here but from our community. It would raise a lot of awareness in our community and people would access. Go and see people from Caribbean culture or the African culture. I think it's just people are just scared sometimes. It requires a lot of years and hard work, and community events and understanding, recruiting from different communities that speak different languages, going to the temple, places of worship. People just didn't want to but now that's opened lots of doors.

## 6 Discussion

Our findings suggest that individuals from SA backgrounds are receptive to DHIs, but identified several barriers and facilitators in accessing and using DHIs, notably linguistic and cultural factors. PPIE participants highlighted the need for interventions to be available in individuals’ preferred languages with culturally tailored content. Being respectful of individual needs and choices, particularly cultural and religious beliefs, and having trusted community leaders promote DHIs were perceived as key to engaging people.

Language was reported to be the biggest barrier, as the majority of online health information is only available in English. People with limited English proficiency are less likely to seek health information online [27], largely due to the scarcity of multilingual websites and patient portals [28]. Although tools, like Google Translate, are increasingly used [29, 30], their accuracy in medical contexts varies greatly [31, 32, 33], and word‑for‑word translation often misses important cultural and linguistic nuances, leading to misunderstandings [34]. Simply translating English content can also alter patients’ perceptions and reported outcomes [34, 35]. To improve accessibility, health information should be produced directly in the target population's languages using professional translators to ensure accuracy and cultural relevance [36]. In addition to language, literacy presents a further challenge. Literacy is an additional obstacle, as many individuals struggle to understand online health information [37], which is often written above the average reading level [36, 38]. Ensuring materials use plain, easy‑to‑understand language is essential to prevent confusion and misinterpretation [27, 39].

Participants reported being more likely to trust, engage with, and act on health information when it is delivered by someone relatable or who shares similar cultural backgrounds or lived experiences. Ensuring accurate and valued representation of diverse populations is essential for reducing health inequalities [40, 41], as a lack of representation can lead to misperceptions about risk and discourage information‑seeking [41]. Peer messengers are viewed as more relatable than healthcare professionals [42], strengthening trust, relevance, and the acceptability and effectiveness of interventions among underserved groups [43, 44, 45]. To improve representation meaningfully, multiple identities should be portrayed in positive, authentic, and non‑stereotypical ways [46, 47], with cultural considerations incorporated at both surface (matching visible characteristics) and deep levels (reflecting shared values, beliefs, and norms) [48].

The complexities of trust were highlighted, noting that engagement with individuals both inside and outside their community can raise fears about confidentiality. Feeling safe to share personal information depended on assurances of privacy and respect. Breaches of confidentiality, particularly by professionals from the same ethnic background, were described as especially damaging to trust. Findings from this PPIE consultation contrast with previous work showing that patients often prefer and trust clinicians of the same racial or ethnic background, reporting higher perceived quality and satisfaction with concordant care [49, 50]. While such preferences are typically linked to shared cultural experiences and culturally informed competence that promote mutual understanding [51], perceptions of trust are also shaped by lived experience and prior encounters with bias [52]. In our consultation, confidentiality emerged as a key determinant of trust, differing from prior studies that emphasised communication, caring, and competence as universal drivers of trust [53]. This suggests that trust may be shaped by context‐specific factors and individual experiences with the healthcare system.

Promotion of DHIs by a trusted person (doctors, peers, faith leaders) was perceived to help alleviate the fear of DHIs and improve uptake and usage. Although healthcare professionals are generally respected, some individuals place greater trust in non‑experts for health information [54]. A multipronged approach that uses trusted messengers and creative communication strategies is therefore important for countering misinformation, building community trust, and supporting widespread engagement. Faith‑based organisations, in particular, play a key role in public health within underserved communities [55], with faith leaders able to adapt health messages in culturally resonant ways and positively influence the health literacy and behaviours of their congregations [56, 57, 58, 59]. Collaborative efforts between healthcare providers and religious leaders can strengthen health promotion, and involving faith leaders in communicating and endorsing DHIs may help overcome mistrust and misinterpretation of online health information [60, 61]. Although online health information is widely available, its reliability varies greatly and can be difficult to judge [27, 62]. Without the skills to critically evaluate sources, people may misinterpret information or rely on low‑quality websites [27, 54, 63, 64]. Evidence shows that scientifically robust content often receives less engagement than lower‑quality material [65], highlighting the need for high‑quality information to be both accessible and engaging [54]. Greater support is needed to help individuals safely navigate online health information, particularly in developing the skills required to identify trustworthy sources.

Digital delivery was widely viewed as an effective way to provide health information, but its impact depends on accessibility, literacy, cultural relevance, and user engagement. Like the participants in Ramasawmy et al. [66] study, our PPIE participants felt that a lack of culturally diverse content reduced engagement with DHIs, even among those without language or access barriers. Culturally adapted interventions consistently outperform non‑adapted ones [67, 68, 69], as adaptation requires more than translation; it must address language, context, concepts, communication, family dynamics, and cultural norms [70]. Meaningful adaptation relies on user involvement [67, 71] to ensure interventions are effective, accessible, acceptable and appropriate for individuals with diverse needs [72, 73]. Co‑producing DHIs with communities facing barriers can enhance engagement, equity of access, and sustained use. Given population heterogeneity, targeted approaches to DHI design and implementation are needed, and mapping user types, barriers, and facilitators, as demonstrated by Ramasawmy et al. [66], can help tailor DHIs for maximum benefit. Flexible co‑design methods can also address barriers such as low literacy, cultural biases, and confidentiality concerns [73]. Insights from the Bridging the Gap project [74] support these findings, and the project's practical guide offers communities a resource for tailoring digital support for South Asian and Black African and Caribbean groups [74, 75]. Collectively, these findings reinforce the need to involve people with diverse demographics, backgrounds, and levels of knowledge in PPIE, as these differences shape user needs and the usability of DHIs.

## 7 Strengths and Limitations

The main limitation of the PPIE consultation was that only a single participant group was interviewed, which limits the generalisability and transferability of the findings. However, the group was relatively large and engaged in three interviews over the study period, enabling in‐depth exploration of perspectives that provide contextual depth and relevance. Given the size of the group, quieter participants may have been overshadowed at times. To mitigate this, the lead researcher, an experienced qualitative researcher, was supported by a community engagement officer who had established relationships with participants, ensuring everyone had the opportunity to contribute. The consultation workshops enabled meaningful patient and public engagement, capturing experiential knowledge that may be overlooked by traditional research; as such, the findings should be interpreted as context‑specific and informative for future research.

A key strength of the consultation was the demographic diversity within the same ethnic group (variations in age, backgrounds, and experiences), which captured variations in perspectives beyond ethnicity, contributing to the richness and depth of the data. The inclusion of multiple perspectives allowed for a more nuanced understanding of the issues discussed. Engaging participants from the same ethnic background likely reduced cross‐cultural misunderstandings and enabled more nuanced exploration of shared cultural perspectives. It may also have made participants feel more comfortable sharing their thoughts, resulting in richer and more authentic data. As this PPIE consultation focused specifically on those from SA backgrounds, the findings may not extend beyond this ethnic group, reducing applicability to other ethnic populations. Further work should explore the perspectives and experiences of DHIs across diverse populations that would allow for cross‐cultural comparisons.

## 8 Conclusion

Our findings described the complex barriers and facilitators to DHIs for people with CKD from SA backgrounds. People from South Asian backgrounds indicated that they are open to DHIs, but emphasised the importance of providing these interventions in their preferred languages and ensuring content is culturally appropriate. Co‐developing DHIs with communities that encounter barriers to healthcare is crucial, as this collaborative approach can enhance engagement, improve equitable access, and support sustained use. These insights will inform adaptations to our existing DHI to better support people with CKD in managing their CKD and overall health.

## Author Contributions

**Courtney J. Lightfoot:** conceptualisation; investigation; funding acquisition; writing – original draft; methodology; writing – review & editing; formal analysis; project administration; supervision; resources. **Roseanne E. Billany:** methodology; writing – review & editing; formal analysis. **Heather Hill:** writing – review & editing. **Matthew P. M. Graham‐Brown:** funding acquisition; conceptualisation; writing – review & editing; methodology.

## Ethics Statement

This work was conducted as Patient and Public Involvement, and thus, ethical approval was not required. All participants provided written informed consent. All participants were given the opportunity to ask questions throughout the consent process.

## Conflicts of Interest

The authors declare no conflicts of interest.

## Data Availability

The data supporting the findings of these PPIE consultations are not publicly available due to privacy and confidentiality agreements with participants. Interview transcripts contain personal information, and while anonymised quotations may be used in publications, full transcripts cannot be shared to ensure participants cannot be identified. Researchers interested in accessing anonymised excerpts may contact the corresponding author and will be required to comply with ethical and confidentiality requirements. Reasonable requests may be made to kidneyhealthPPIE@leicester.ac.uk.
